# Tribute to Professor Stefan Kunz

**DOI:** 10.3390/v13091862

**Published:** 2021-09-18

**Authors:** Manuel Pascual

**Affiliations:** 1 Faculty of Biology and Medicine, University of Lausanne, 1011 Lausanne, Switzerland; Manuel.Pascual@unil.ch; 2 Lausanne University Hospital, 1011 Lausanne, Switzerland

It has been a year since Stefan Kunz, a Full Professor since 2017 at the Faculty of Biology and Medicine of the University of Lausanne, Switzerland, passed away. His death was a shock and an enormous loss, not only for his family and friends, but also the entire Swiss and international scientific community, and especially our Faculty of Biology and Medicine and the University Hospital Center of Lausanne (CHUV). Professor Kunz always impressed us for being a deeply engaging, charismatic and gifted teacher, and for his benevolence, his energy and his discoveries.

We miss Professor Kunz, who specialized in the discipline of emerging viruses, focusing his efforts on understanding how viruses succeed in breaking species barriers to then hijack the human host cell machinery.

He started his career in the field of neurosciences, earning his PhD thesis at the University of Zurich, with a special interest in neural signaling. He then went to the Scripps Research Institute in California as a postdoctoral fellow, where he worked with one of the greatest experts in virology worldwide, Michael Oldstone. He worked in San Diego for almost 10 years, rising to the rank of Associate Professor at the Scripps Research Institute. Returning to Switzerland in 2008, he joined the Institute for Microbiology of the University of Lausanne.

His contributions are invaluable on every level: first and foremost, through his research on hemorrhagic fever viruses, dangerous pathogens for mankind, with his special focus on Lassa Virus. Furthermore, he produced excellent work on transmission and infection with emerging viruses such as Ebola.

As a mentor, he also created a veritable “school” of virology in Lausanne, involving dozens of researchers. He conceptualized new courses, conveying his own philosophy of teaching, his passion for medicine and his curiosity for research. Stefan Kunz was an extraordinary colleague and personality, and he will forever remain in our hearts and our memory. We would like to honor Professor Kunz and celebrate all that he has given us.

